# The −149C>T polymorphism of DNMT3B is not associated with colorectal cancer risk: Evidence from a meta-analysis based on case-control studies

**DOI:** 10.3892/etm.2012.638

**Published:** 2012-07-17

**Authors:** CHUNYAN FANG, WENQI SUN, HUIRONG HAN, LIHONG SHI, LIN WANG, YAN ZHAO, YANG TAN

**Affiliations:** 1 Laboratory of Applied Pharmacology, Weifang Medical University, Weifang 261042;; 2 The Fifth People’s Hospital of Weifang, Weifang 261000;; 3 Department of Cardiology, The Second Hospital of Tianjin Medical University, Tianjin Institute of Cardiology, Tianjin 300211;; 4 Digestive Department of Chongqing First People’s Hospital, Chongqing 400011, P.R. China

**Keywords:** −149C>T polymorphism, DNA methyltransferase 3B, colorectal cancer risk, meta-analysis

## Abstract

The aim of this study was to examine the association between the −149C>T polymorphism of DNA methyltransferase 3B (DNMT3B) and colorectal cancer (CRC) susceptibility. A comprehensive search was conducted to identify all case-control studies of the −149C>T polymorphism of DNMT3B and CRC risk. Statistical analysis was performed with the software program Stata (version 12.0) and Review Manager (version 5.0). A total of seven eligible studies, including 2,666 cases and 4,022 controls, associating the DNMT3B polymorphism of −149C>T with the risk of CRC were identified. These studies suggested no significant associations between the −149C>T polymorphism of the DNMT3B gene and the risk of developing CRC in the recessive, dominant and co-dominant models [for CC vs. TT: odds ratio (OR), 0.90; 95% confidence interval (CI), 0.90–1.25; P=0.37; for the recessive model: OR, 0.54, 95% CI, 0.28–1.04; P<0.00001; for the dominant model: OR, 1.07; 95% CI, 0.93–1.23; P=0.83 and C allele vs. T allele: OR, 0.70; 95% CI, 0.43–1.13; P<0.00001]. In the subgroup analysis, no significant associations were found in the European populations (for CC vs. TT: OR, 1.09; 95% CI, 0.92–1.30; P=0.88; for the recessive model: OR, 1.00; 95% CI, 0.88–1.13; P=0.14; for the dominant model: OR, 1.50; 95% CI, 0.89–2.54; P<0.00001 and C allele vs. T allele: OR, 0.70; 95% CI, 0.38–1.28; P<0.00001). No significant association was found between the −149C>T polymorphism in DNMT3B and CRC susceptibility.

## Introduction

Colorectal cancer (CRC) is a worldwide public health problem, resulting in approximately 500,000 mortalities every year (1). Previous studies demonstrated that colorectal carcinogenesis is a complicated multi-step process involving changes of numerous oncogenes and tumor suppressor genes induced by the interaction of various factors. Simultaneously, other factors, including high alcohol intake, low methionine, low folate diet, smoking status and environmental carcinogenic agents are assumed to be possible risk factors of CRC. DNA aberrant methylation may also increase the risk of CRC (2,3). Not all individuals exposed to the above exogenous risk factors develop CRC, which indicates that the individual susceptibility factors may play a key role in cancer development. DNA methylation is a major epigenetic mechanism that regulates chromosomal stability and gene expression in mammalian cells (4,5). Aberrant DNA cytosine methylation may play a key role in carcinogenesis since methylation facilitates gene mutation via the deamination of 5-methylcytosine to thymine (6). DNA methyltransferase 3B (DNMT3B) is required for the establishment and maintenance of genomic methylation patterns and proper murine development (7). It is upregulated in certain malignancies, including bladder, pancreatic, kidney and colon cancer (8).

To date, no meta-analysis has been conducted to investigate the association between the −149C>T polymorphism of DNMT3B and CRC. Thus, a meta-analysis based on a total of seven independent studies was performed to determine whether there was any evidence of a correlation between the DNMT3B −149C>T polymorphism and CRC susceptibility.

## Materials and methods

### Publication search

We searched the articles using the terms ‘DNMT3b’, ‘DNMT3B’, ‘C46359T’, ‘−149C>T’, ‘rs2424913’, ‘neoplasm’, ‘carcinoma’, ‘tumor’, ‘variation’, ‘colorectal’ and ‘cancer’ in the electronic databases MEDLINE, ISI Web of Knowledge and Embase, without date and language restrictions, and all eligible studies were obtained prior to March 21, 2012. We evaluated the associated literature to retrieve the most eligible studies. The reference lists were hand-searched to find other relevant studies. Of the studies with overlapping data published by the same investigators, only the most recent or complete study was included in this meta-analysis.

### Inclusion and exclusion criteria

The following inclusion criteria were used to select studies for this meta-analysis: i) only case-control studies were considered; ii) the study was required to describe CRC diagnoses and the sources of cases and controls and iii) the authors had to provide the size of the sample, odds ratio (OR) and their 95% confidence interval (CI) or the information that infers the results in the studies. Reviews and bibliographies of the relevant studies, and references of all the included studies were also hand-searched. The exclusion criteria were: i) non case-control studies; ii) a control population including malignant tumor patients and iii) duplicated studies.

### Data extraction

Two investigators reviewed and extracted information from all eligible studies independently, according to the inclusion and exclusion criteria listed above. An agreement was reached by discussion between the two reviewers whenever there was a conflict. The following items were collected from each study: first author’s surname, year of publication, statistical data, ethnicity, total number of cases and controls as well as numbers of cases and controls with CC, CT and TT genotypes. Various ethnicities were classified as European, Asian and mixed populations.

### Statistical analysis

The effect measure of choice was OR with corresponding 95% CI. The significance of the summary OR was determined with a Z-test and P<0.05 was considered to indicate a statistically significant result. In the present study, two models of meta-analysis were applied for dichotomous outcomes; the fixed and random effects models. The fixed effects model assumes that studies are sampled from populations with the same effect size, making an adjustment to the study weights according to the in-study variance. The random effects model assumes that studies are obtained from populations with varying effect sizes by calculating the study weights both from the in-study and between-study variances, considering the extent of variation or heterogeneity. Heterogeneity assumption was checked by the Q-test. P≥0.10 for the Q-test indicated a lack of heterogeneity among the studies.

First, we examined −149C>T genotypes using additive (CC vs. TT), recessive (CC vs. CT+TT) and dominant (CC+CT vs. TT) genetic models. The comparison of the C allele with T allele was then examined. Funnel plots were produced and an asymmetric plot indicates there is no publication bias. The symmetry of the funnel plot was then evaluated by Egger’s linear regression test. The significance of the intercept was determined by the t-test suggested by Egger (P<0.05 was considered to indicate a statistically significant publication bias). Statistical tests were performed with Review Manager (version 5.0) and Stata (version 12.0) using two-sided P-values.

## Results

### Eligible studies

Seven studies on DNMT3B −149C>T geno-types and CRC were identified through the literature search and selection based on the inclusion and exclusion criteria (8–14). The seven independent studies consisted of four European, two Asian and one mixed population. In total, 2,666 CRC cases and 4,022 controls were included in the meta-analysis. The selected study characteristics are shown in Table I.

### Meta-analyses and evaluation of heterogeneity and publication bias

The meta-analysis of the association of the −149C>T polymorphism of DNMT3B with CRC in an overall population included seven independent studies with a total of 2,666 cases and 4,022 controls. The Q-test of heterogeneity was significant and we conducted analyses using fixed and random effects models.

**Figure 1:**
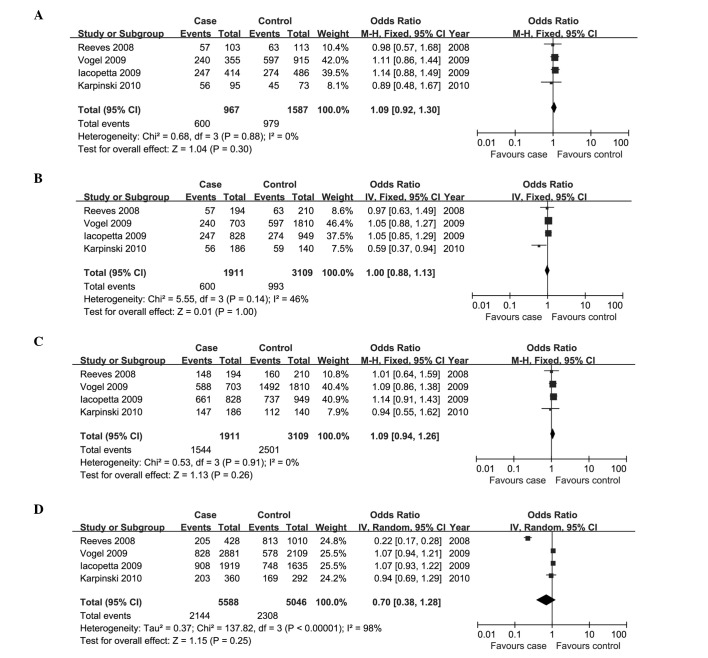
Forest plots of odds ratios (OR) with 95% confidence intervals (CI) for DNA methyltransferase 3B (DNMT3B)−149C>T polymorphisms and risk of colorectal cancer in European population. (A) CC vs. TT, (B) CC vs. CT+TT, (C) CC+CT vs. TT, (D) C allele vs. T allele.

**Figure 2:**
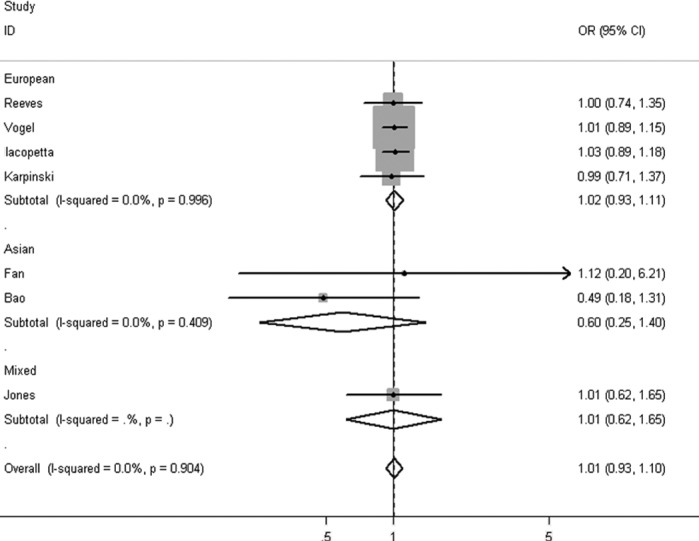
Forest plot of colorectal cancer risk associated with the DNMT3B −149C>T polymorphism (for CC+CT vs. TT). The squares and horizontal lines correspond to the study-specific OR and 95% CI. The area of the squares reflects the weight (inverse of the variance). The diamond represents the summary OR and 95% CI.

**Figure 3:**
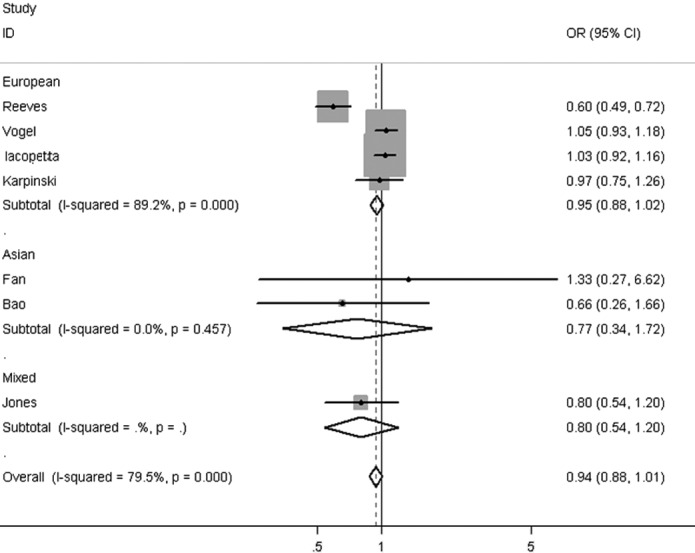
Forest plot of colorectal cancer risk associated with the DNMT3B −149C>T polymorphism (for C allele vs. T allele). The squares and horizontal lines correspond to the study-specific OR and 95% CI. The area of the squares reflects the weight (inverse of the variance). The diamond represents the summary OR and 95% CI.

No statistically significant difference was found in CRC risk between the patients with CC genotype and those with TT genotype (OR, 0.90; 95% CI, 0.90–1.25; P=0.37). Similarly, no significant associations were found in the recessive model (CC vs. CT+TT) comparison (OR, 0.54; 95% CI, 0.28–1.04; P<0.00001) or dominant model (CC+CT vs. TT) comparison (OR, 1.07; 95% CI, 0.93–1.23; P=0.80). In addition, we did not detect any association between the DNMT3B −149C>T polymorphism and CRC when examining the contrast of C vs. T alleles (OR, 0.70; 95% CI, 0.43–1.13; P<0.00001). In the stratified analysis by ethnicity, significant between-study heterogeneity was detected in all the comparisons in Europeans, but not in Asian and mixed populations. For the European population, there was no significant association between the −149C>T polymorphism and CRC in the additive model comparison (OR, 1.09; 95% CI, 0.92–1.30; P=0.88; Fig. 1A), recessive model comparison (OR, 1.00; 95% CI, 0.88–1.13; P=0.14; Fig. 1B), dominant model comparison (OR, 1.50; 95% CI, 0.89–2.54; P<0.00001; Fig. 1C) and C vs. T alleles comparison (OR, 0.70; 95% CI, 0.38–1.28; P<0.00001; Fig. 1D). Simultaneously, in the stratified analysis for −149C>T polymorphism, we did not find any associations between the −149C>T polymorphism and CRC risk in Asian and mixed populations (data are shown in Table II, Figs. 2 and 3).

Begg’s funnel plot and Egger’s test were performed to assess the publication bias. The results did not show any evidence of publication bias in any of the comparisons.

## Discussion

A single nucleotide polymorphism (SNP) is the most common form of human genetic variation, and may contribute to susceptibility to cancer, however, the underlying molecular mechanism is unknown. Previous studies suggested that certain variants, especially those in the promoter regions of genes, may affect either the expression or activity levels of enzymes (15–17) and therefore may be mechanistically associated with cancer risk. The DNMT3B gene, located on chromosome 20q11.2, contains several SNPs. It was assumed that the DNMT3B SNP may modify susceptibility to several types of tumors (17–20), including lung, breast, colon and CRCs. Previous studies on the correlation between DNMT3B polymorphisms and CRC risk were contradictory. These inconsistent results are possibly due to a small effect of the polymorphism on CRC risk or the relatively low statistical power of the published studies. Thus, the meta-analysis was required to provide a quantitative approach for combining the results of various studies with the same topic, and for estimating and explaining their diversity.

The present meta-analysis, including 2,666 cases and 4,022 controls, concerning the −149C>T polymorphism of DNMT3B gene and CRC risk. In the meta-analysis, we did not find that the variant genotypes of the DNMT3B −149C>T polymorphisms were significantly associated with CRC risk. Simultaneously, the same results were observed in a stratified analysis by ethnicity.

In this study, we have found that the variant genotype of the DNMT3B −149C>T polymorphism, in the European population, was not associated with a significant increase in CRC risk. This result was consistent with previous studies (9,21). Although the DNMT3B −149C>T polymorphism may be associated with DNA repair activity, no significant association of the variant genotype with CRC risk was found in European and Asian populations, suggesting that the effect of the genetic variant may be masked by the presence of other unidentified causal genes involved in CRC.

Certain limitations of this meta-analysis should be acknowledged. First, the number of cases and controls in the included studies was not sufficient. Second, our result was based on unadjusted estimates, while a more precise analysis should be conducted and other factors including diet, smoking status, drinking status and environmental factors should be considered. Third, in the subgroup analyses by ethnicity, a relatively limited number of studies available made it impossible to perform ethnic subgroup analysis of Asians and mixed populations. Thus, additional studies are required to evaluate the effect of this functional polymorphism on CRC risk in different ethnicities, particularly in Asians. In addition, our analysis did not consider the possibility of gene-gene or SNP-SNP interactions or the possibility of linkage disequilibrium between polymorphisms.

In conclusion, this meta-analysis provided evidence of the association between the −149C>T polymorphisms and CRC risk, supporting the hypothesis that −149C>T polymorphisms were not correlated with overall CRC risk. In the subgroup analysis, the same results were found in European, Asian and mixed populations. To verify our findings, larger and well-designed studies are required to evaluate the association between the DNMT3B −149C>T polymorphism and CRC risk.

## Figures and Tables

**Table I:** Characteristics of the primary studies included in the meta-analysis.

Study	Year	Ethnicity	Genotyping method	Sample size cases/controls	Genotype distribution of cases/controls		
					CC	CT	TT
Jones *et al* (9)	2006	Mixed	PCR-RFLP	74/72	12/28	45/27	17/17
Fan *et al* (10)	2008	Asian	PCR-RFLP	137/308	0/0	2/4	135/404
Reeves *et al* (11)	2008	European	PCR-RFLP	194/210	57/63	91/97	46/50
de Vogel *et al* (12)	2009	European	PCR-RFLP	703/1810	240/597	348/895	115/318
Iacopetta *et al* (13)	2009	European	PCR-RFLP	828/949	247/274	414/463	167/212
Karpinski *et al* (14)	2010	European	PCR-RFLP	186/140	56/45	91/67	39/28
Bao *et al* (8)	2011	Asian	PCR-RFLP	544/533	0/0	6/12	538/521

PCR-RFLP, polymerase chain reaction-restriction fragment length polymorphism.

**Table II:** Characteristics of the primary studies included in the meta-analysis.

Studies	Cases/controls	CC vs. TT		CC vs. CT+TT		CC+CT vs. TT		C/Tallele	
		OR (95% CI)	P-value	OR (95% CI)	P-value	OR (95% CI)	P-value	OR (95% CI)	P-value
Total (7)	2666/4022	0.90 (0.90–1.25)	0.37	0.54 (0.28–1.04)	<0.00001	1.01 (0.93–1.10)	0.80	0.70 (0.43–1.13)	<0.00001
European (4)	1911/3109	1.09 (0.92–1.30)	0.88	1.00 (0.88–1.13)	0.14	1.50 (0.89–2.54)	<0.00001	0.70 (0.38–1.28)	<0.00001
Asian (2)	681/841	NA	NA	NA	NA	0.59 (0.25–1.39)	0.40	0.63 (0.27–1.48)	0.26
Mixed (1)	74/72	0.67 (0.29–1.51)	NA	0.42 (0.2–0.88)	NA	1.01 (0.62–1.65)	NA	0.80 (0.54–1.20)	NA

NA, not available.
